# Evaluating external load responses to cumulative playing time and position in the European Handball Federation Women’s Euro 2022 through an IoT and Big Data architecture approach

**DOI:** 10.5114/biolsport.2025.144409

**Published:** 2024-10-25

**Authors:** Claude Karcher, Roger Font, Diego Marcos-Jorquera, Virgilio Gilart-Iglesias, Carmen Manchado

**Affiliations:** 1 Biomedicine Research Center of Strasbourg (CRBS), Mitochondria, Oxidative Stress, and Muscle Plasticity Laboratory (UR 3072), University of Strasbourg, Strasbourg, France; 2 European Centre for Education, Research and Innovation in Exercise Physiology (CEERIPE), Faculty of Sport Sciences, University of Strasbourg, Strasbourg, France; 3 CREPS de Strasbourg, 67200 Strasbourg, France; 4 Research group in Tecnologia Aplicada a l’Alt Rendiment i la Salut (TAARS), Tecnocampus, Department of Health Sciences, Pompeu Fabra University, Mataró, Spain; 5 GRCE Research Group, National Institut of Physical Education of Catalonia (INEFC), Barcelona, Spain; 6 Department of Computer Science and Technology, Polytechnic School, University of Alicante, San Vicente del Raspeig, Spain; 7 Physical Education and Sports, Faculty of Education, University of Alicante, San Vicente del Raspeig, Spain; 8 European Handball Federation, Methods Commission, Vienna, Austria

**Keywords:** Women’s, Female, Team sport, Load, Fatigue development, IMUs

## Abstract

The quantification of physical demands placed upon handball players, segmented by their specific roles and duration of play, is crucial for sustaining high performance and minimizing the risk of injury. Leveraging advanced inertial measurement units, this investigation captured and analyzed the external load data of athletes participating in the EHF Women’s EURO 2022. The aim of this study was to provide coaching staff with information on fatigue development during periods of high match density. The study evaluated the effects of playing position and cumulative playing time on external load metrics, using linear mixed models that treated individual players as random effects. The study employed a cutting-edge computational framework integrating sensor network technologies, Local Positioning Systems (LPS), and Big Data Analytics within a descriptive analytics methodology. From over half a billion raw records, we distilled 1,013 data entries from 47 matches for analysis. The findings reveal that the wings demonstrated the highest levels of total and high-speed running distances, though they sustained lower PlayerLoad relative to backs. Interestingly, cumulative playing time did not markedly alter load profiles, which may be attributed to strategic substitution decisions by coaches and the players’ own pacing strategies. Notable discrepancies within positional demands were observed over time, such as centers displaying increased distance coverage within the 2–3 hour play interval. This study underscores the efficacy of strategic load management and tailored pacing in sustaining player performance throughout high-stakes tournaments. It elucidates the relationship between managerial tactics and player-specific characteristics in the context of external load distribution.

## INTRODUCTION

Mastering player-specific demands in competition, particularly regarding their playing position, is increasingly vital in modern team sports [1]. Upon understanding these needs, it is crucial to analyse whether the training during the microcycle adequately prepares players to meet these demands [2]. Such insights are key for technical staffs to be able to plan effective training strategies, enhancing the overall sporting performance of their teams [2].

Inadequate management or control of sport-specific demands and training loads has been shown to increase the risk of injury [3], and can lead to a decline in the performance of individual players or of the team as a whole [4]. Therefore, it is essential to monitor training load at both internal and external levels, as a single variable cannot determine the injury rate or the fitness level of the players [3].

Recent studies using advanced IMUs devices have yielded accurate and ecologically valid data in handball research. This technology has been effectively employed in men’s handball in official matches [1, 2, 5–9] and team training sessions [2] providing insight without disrupting their competition or training dynamics. This technology has been validated by previous studies [10, 11] and proven to be highly effective for handball, significantly enhancing the reliability and precision od data regarding players’ physical performance and physiological states.

Upon reviewing the research conducted with this or similar technology to evaluate the external load women’s handball players endure, it is apparent that the output is significantly lower when compared to research with male players [12]. This research tells us that female players run an average of 2071.0 ± 1048.5 m [9] and if we analyse it by position, the wings are the ones who accumulate more distance. If we look in detail at these metres covered by the wings, we can see that the left wing runs 515.6 ± 245.1 m in fast running and 170.4 ± 101.6 m in sprinting. The right wing covered 507.2 ± 225.1 m in fast running and 150.6 ± 84.9 m in sprinting [9].

Regarding the frequency of high-intensity accelerations and decelerations per minute, and using a video-based system, it emerges that the back players accumulate the most [13]. However, upon further analysis of high-intensity accelerations and decelerations per minute, it becomes evident that the wing players achieve the highest values [6].

Regarding PlayerLoad, players can accumulate 418.3 ± 141.2 a.u. per match [7]. Upon analysing this variable in relation to time, it is evident that consensus is lacking with some studies indicating that pivots accumulate the greatest external load [14], while others suggest it could be the wings [15].

Beyond understanding training and match demands, another critical aspect is the recovery of players between matches or training sessions, which requires effective fatigue management [16]. Incomplete recovery from accumulated fatigue can lead to increased injury risk or diminished sport performance [3, 4].

The current trend in sports, especially in football, shows an increase in weeks with higher competitive density at both club [17] or national team competitions [18]. This increased frequency makes proper recovery difficult increasing more challenging heightening the risk of injury [18, 19]. In football, research indicates that during periods of high match density, players with more minutes on the field show lower external load levels compared to those with less playing time [20] or lower values at the average level [19].

Another research in football with young players, analysed the data in periods of 15 minutes and it was observed that the players modulated the intensity of their actions since no differences were observed at the maximum level, but at the average level in terms of external load [21]. It could be said that players seek their strategies to be able to maintain the optimal level in high intensity actions by maintaining the maximum intensity and reducing the intensity of the actions that are not so demanding [4].

In women’s sports, research indicates that injuries are more prevalent in national team tournaments with a higher match density compared to club competitions [22]. Additionally, during periods of match congestion, a decline in the performance of female football players has been noted [23]. In hockey, a reduction in muscle strength has been documented [24], potentially affecting neuromuscular control, increasing the risk factors for injury and elevating injury risk, especially to the knee [23].

Investigating indoor sports reveals that match congestion significantly impacts player and team performance [25] leading to diminished effectiveness of technical elements [17] or a reduced intensity [4]. It is important to highlight that in international tournaments, such as the EHF Women’s EURO 2022, teams reaching the finals, faced a total of 8 matches within 15 days, encountering a very dense schedule.

This study aims to provide coaches and technical staff with comprehensive insights into fatigue development during periods of high match density, like the EHF Women’s EURO which can lead to technical-tactical errors and increase the risk of injury. It focuses on understanding the effects of playing time on external load demands across diverse playing positions.

The emergence of the Internet of Things (IoT) and Big Data technologies offers new opportunities to enhance our understanding of player performance and fatigue management. In recent years, these technologies have been increasingly adopted in team sports research, demonstrating their effectiveness in providing real-time feedback, enhancing training load management, and improving overall performance.

IoT refers to a network of interconnected devices that collect and transmit data in real time. In sports science, IoT devices, such as wearable sensors and GPS trackers, can gather continuous, detailed data on various physiological and biomechanical parameters without interfering with the athlete’s natural movements. These devices provide valuable insights into players’ external and internal load, enabling more precise monitoring and individualized training programs. Big Data, on the other hand, involves the storage, processing, and analysis of vast amounts of data from diverse sources. In the context of sports, Big Data analytics allows for the integration and examination of data collected from IoT devices, video analysis, and other monitoring tools. This comprehensive approach enables coaches and sports scientists to identify patterns, predict injury risks, and optimize performance based on data-driven decisions, even more so in collective sports with many player changes such as handball.

To address these gaps and enhance our understanding of external load demands and fatigue management in women’s handball, this study introduces an innovative approach using IoT and Big Data architecture. The application of IoT devices allows for real-time, precise data collection regarding player performance, providing insights that traditional methods cannot achieve. Furthermore, Big Data analytics enables the handling and analysis of vast datasets generated by IoT devices, facilitating data-driven decision-making to optimize training loads and reduce injury risks. This combination of IoT and Big Data is particularly effective in high-density competition periods, offering a comprehensive perspective on player fatigue and recovery that can significantly inform coaching strategies and enhance team performance.

## MATERIALS AND METHODS

### Experimental approach to the problem

We analysed the external load data obtained in the European Handball Federation (EHF) Women’s EURO held in Slovenia, North Macedonia and Montenegro from 4 to 20 November 2022. The championship was contested by 16 national teams and a total of 47 matches were played. Senior handball matches consist of two 30’ halves with a 15’ break in between. The teams are made up of 16 players playing a maximum of 7 players at a time on the field. The positions of the players are Goalkeeper (GK), Left Wing (LW), Right Wing (RW), Left Back (LB), Right Back (RB), Centre Back (CB) and Pivots (PIV).

A total of 284 players (175.3 ± 11.1 cm height, 71.9 ± 8.9 kg body mass) took part in the study, 43 CB (172.5 ± 12.0 cm height, 70.5 ± 16.8 kg body mass), 47 LB (179.1 ± 7.9 cm height, 73.8 ± 5.5 kg body mass), 39 LW (169.1 ± 12.4 cm height, 65.6 ± 4.4 kg, 44 LP (179.8 ± 8.4 cm height, 78.6 ± 4.6 kg body mass), 35 RB (177.5 ± 73.9 cm height, 73.9 ± 5.5 kg body mass), 33 RW (168.9 ± 10.3 cm height, 65.5 ± 58 kg body mass) and, 43 GK (178.3 ± 109 cm height, 73.9 ± 4.2 kg body mass).

The research data emerged thanks to the daily monitoring process of the players during matches; therefore, relevant approval of the ethics committee was not required [26]. Nonetheless, the study was conducted following the ethical principles for biomedical research with human beings, established in the Declaration of Helsinki of the World Medical Association (updated in 2013), and the club’s managerial structure approved its implementation.

**FIG. 1:**
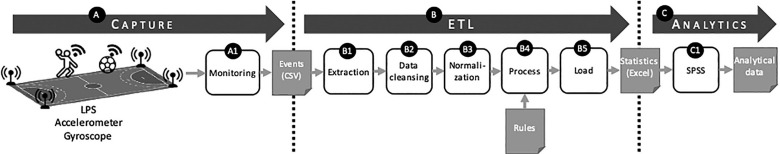
Methodology for data analysis.

In order to obtain and analyse the information, a comprehensive system based on a set of IoT sensors network, LPS and big data analytics was designed following the methodology described in Figure 1.

For this study, 526,579,944 records were analyzed in regards to accelerations, decelerations, impacts and jumps.

### Games Monitoring (Figure 1, Capture Layer – IoT system)

The study was carried out using the IoT Kinexon system (Kinexon SafeTag, Kinexon Precision Technologies, Munich, Germany). Each device, whose dimensions were 49 × 33 × 8 mm (height/width/depth) and weighed 14 g, was fitted to the back of each player with an adjustable vest.

The device provides 9-axis inertial data (accelerometer, gyroscope, magnetometer) capable of recording accelerations/decelerations, rotations and orientation angles with a refresh rate of up to 60 Hz. The device has been validated [10] and used for handball movement time analysis in handball [1, 8, 9]. The Kinexon system works by triangulations between 9 antennas located around the handball court and connected to a server, and 10 reference antennas acting as anchors.

During games, playing time was only recorded when the players were on the court. The time spent between player rotation, Team Time Outs (a maximum of three per team with a maximum of six per match), periods when the game was interrupted, and the disciplinary sanctions typical of handball, where players must leave the court for two minutes, were omitted.

### Data processing (Figure 1, ETL layer in Big Data system)

For our study, we calculated the variables of interest shown in table 1. Subsequently, we aggregated the playing time for each player and categorized them into intervals of cumulative playing time within the competition: 0 to 1 hour, 1 to 2 hours, 2 to 3 hours, 3 to 4 hours, 4 to 5 hours, 5 to 6 hours, and over 6 hours. To compare the result, we normalized the variable per minute played.

**TABLE 1:** List and characteristics of the variables analysed

VARIABLES	UNITS	DEFINITION
Total Distance Covered (TD)	Meters (m)	Total distance covered by the player during the match based on the player’s LPS data

High-speed running (HSR)	Meters per second (m/s)	Total meters travelled (≥ 4.4 m/s)

Number of High-Intensity Actions (HIA)	Arbitrary units (a.u.)	Sum of different variables: Jump > 0.2 m, Impacts > 5 g, Accelerations > 2 m/s^2^, Decelerations > 2 m/s^2^, and shots > 22 m/s

PlayerLoad (PL)	Arbitrary units (a.u.)	The accumulated square root of the sum of the squared instantaneous rates of acceleration changes in each one of the three planes $Plyr.Ld{\left(acc\right)}_{t=n}=\sum_{t=0}^{t=n}\sqrt{\left((fwd_{t=i+1}-fwd_{t=1}{)}^{2}+(side_{t=i+1}-side_{t=1}{)}^{2}+(up_{t=i+1}{)}^{2}\right)}$ For *t* = 0,0.01,0.02,0.03…*n*

Playing time per match	Minutes (min)	Total minutes played in the match

Playing Position	Goalkeepers (GK) Left Wing (LW) Right Wing (RW) Left Back (LB) Right Back (RB) Center Back (CB) Pivot (PIV)	Indicated by the team roster

Our initial dataset comprised 1,378 entries. In the data cleaning process, we excluded players who participated for less than 10 minutes per match and entries with erroneous data (e.g., substantial playing time recorded without corresponding distance covered). Additionally, we removed players from the four teams eliminated in the first round (Switzerland, North Macedonia, Poland and Serbia) as they did not contribute to the research focus, which centred on the accumulation of fatigue. Following these criteria, our dataset was reduced to 1,013 entries.

Finally, in the loading process (Figure 1 – B5), all the information was transformed into Excel format files, compatible with the Statistical Package for Social Sciences (SPSS V22.0 for Windows, SPSS Inc, Chicago, USA) software to perform statistical analysis.

### Statistical analysis (Figure 1, Analytic Layer in Big Data system)

To investigate the impact of playing position and cumulative playing time on players’ performance metrics—total distance covered, high-speed running distance, high-intensity actions, and PlayerLoad—we hypothesized that these factors would significantly influence performance outcomes. To address the repeated measures and nested data structure characteristic of sports performance analysis, linear mixed-effects models were utilized. We constructed four separate models, one for each dependent variable. Playing position and cumulative playing time intervals were included as fixed effects, while players were modelled as a random effect. This approach acknowledges that individual players possess unique, unobserved attributes that influence their performance beyond their playing position or the duration of play. Incorporating players as random intercepts allowed us to capture within-player variability and control for the non-independence of observations from the same player, thereby providing more precise fixed effect estimates. To determine the effect of cumulative playing time within playing positions, we calculated effect size. The t-statistics and degrees of freedom (df) from the mixed models were converted to provide effect-size correlations (r), which were interpreted as trivial (0.2), small (0.2–0.6), moderate (0.6–1.2), large (1.2–2.0), and very large (2.0–4.0) [27]. The 95% confidence intervals for these effect sizes were also calculated to assess their precision. Normality assumptions were verified through visual inspection of histograms for the raw data and examination of model residuals using Quantile-Quantile (Q-Q) plots. The majority of points in the Q-Q plots conformed to the reference line, suggesting an approximate normal distribution of residuals and supporting the suitability of our statistical models. To know if playing time interval had an effect within playing position, we calculate pair wise standardized difference between the different playing time interval. Considering the huge number of results, we decide to report only the effect size above 0.8 with a 90% lower limit above 0.2. The data analysis was performed using the lmer function from the R “lme4” package and the “effsize” package, facilitated by RStudio software (version 2023.12.0).

## RESULTS

**FIG. 2:**
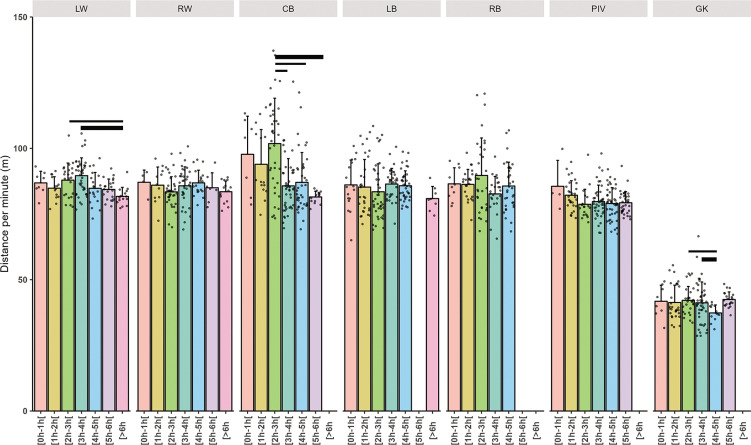
Mean ± SD total distance covered per minute related to total time player and playing positions (Left Wing (LW), Right Wing (RW), Center Back (CB), Left Back (LB), Right Back (RB), Pivot (PIV), and Goalkeeper (GK)). The thickness of the horizontal bar is proportional to the effect size magnitude, with thicker bars denoting a very large effect and thinner bars a large effect.

**FIG. 3:**
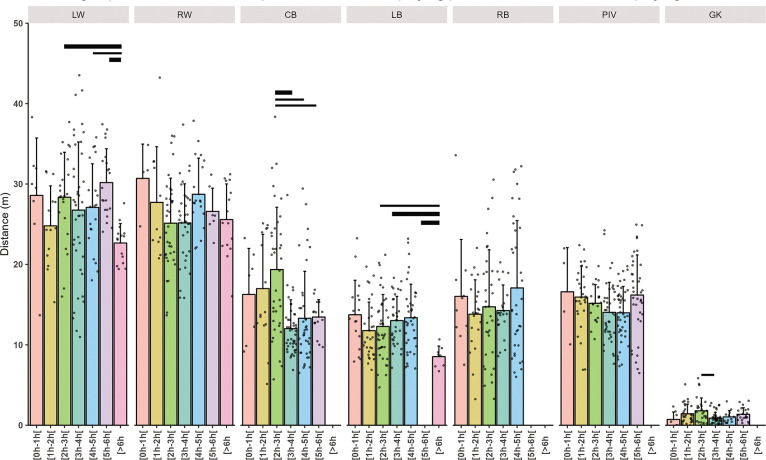
Mean ± SD high-speed distance related to total time player and playing positions (Left Wing (LW), Right Wing (RW), Center Back (CB), Left Back (LB), Right Back (RB), Pivot (PIV), and Goalkeeper (GK)). The thickness of the horizontal bar is proportional to the effect size magnitude, with thicker bars denoting a very large effect and thinner bars a large effect.

**FIG. 4:**
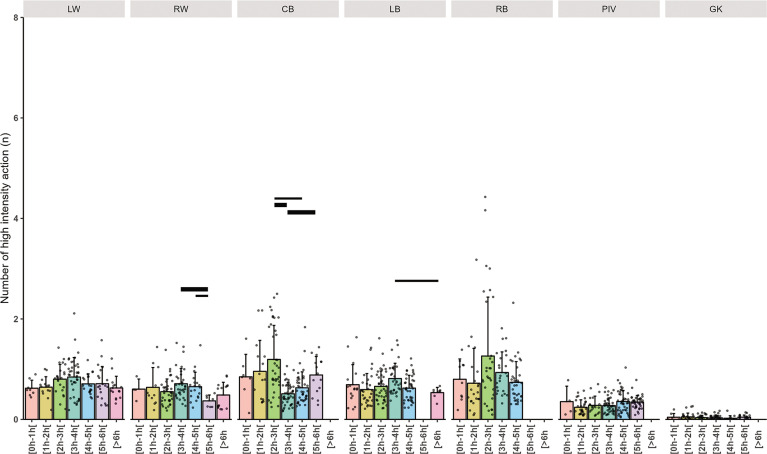
Mean High intensity actions per minute related to total time player and playing positions (Left Wing (LW), Right Wing (RW), Center Back (CB), Left Back (LB), Right Back (RB), Pivot (PIV), and Goalkeeper (GK)). The thickness of the horizontal bar is proportional to the effect size magnitude, with thicker bars denoting a very large effect and thinner bars a large effect.

**FIG. 5:**
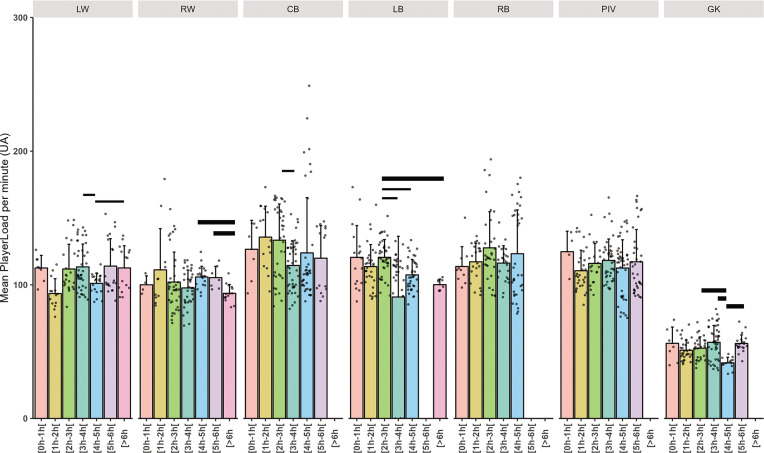
Mean PlayerLoad per minute related to total time player and playing positions (Left Wing (LW), Right Wing (RW), Center Back (CB), Left Back (LB), Right Back (RB), Pivot (PIV), and Goalkeeper (GK)). The thickness of the horizontal bar is proportional to the effect size magnitude, with thicker bars denoting a very large effect and thinner bars a large effect.

Individual and mean total distance covered per minute related to playing position are presented in figure 2, high-speed running distance covered per minute in figure 3, high intensity action per minute in figure 4 and PlayerLoad in figure 5.

**Total Distance Covered:** GKs exhibited a very large decrease in total distance covered (-45.75 m/mn, p < 0.001), while CBs increased their total distance significantly compared to the reference position, albeit with a small ES (Estimate: 4.03, ES: 0.14, p = 0.033). A trivial but significant decrease in total distance was seen in players with cumulative playtimes of 4 to 5 hours (Estimate: -3.68 m/mn, ES: -0.13, p = 0.038). When considering the within playing position differences between cumulative playing time interval, CBs had up to large differences for the 2–3 hours vs. the other time interval (ES ranged from 1.01 to 1.38). The players that played more than 6 hours and 5 to 6 hours in LW and GK respectively covered up to largely less distance than the other cumulative time frame (ES: -1.02 to -1.73 and 1.33 to 1.12 respectively).

**High-Speed Running (HSR) Distance:** The average high-speed running distance was set at 28 m/mn (p < 0.001). GKs again showed a very large decrease (-25.79 m/mn, ES: -1.46, p < 0.001). Players in the 6 to 8 hours and 3 to 4 cumulative playtime group had trivial but significant decrease in high-speed running distance (-4.32 m/mn, ES: -0.14, p = 0.031 and -1.95 m/mn, ES: -0.13 p = 0.04). When examining the differences within playing positions across cumulative playing time intervals for high-speed running distance, LWs and LBs in the 6 to 8-hours’ time frame covered a very large reduced distance (ES: 1.05 to 2.09 for LWs and 1.01 to 1.61 for LBs), whereas CBs in the 2 to 3-hours’ time frame covered a significantly larger distance (ES: 0.8 to 1.25).

**High-Intensity Actions (HIA):** The base rate for high-intensity actions was 2.90 actions per minute (P < 0.001). GKs had a large reduction in high-intensity actions (HIA/mn, ES: -0.80, p < 0.001). No clear effect of the cumulative playtimes was found. The differences within playing positions across cumulative playing time intervals showed that RWs in the 5 to 6-hours’ time frame and CBs in the 3 to 4-hours’ time frame showed up a very large decrease in HIA action performed per minute (ES: 1.04 to 1.41). **PlayerLoad:** An average PlayerLoad value of 111.15 AU was identified (p < 0.001). GKs showed a moderate decrease in PlayerLoad (-54.21 AU, ES: -0.72, p < 0.001). A small decrease in PlayerLoad was observed for players with cumulative playtimes between 4 to 5 hours (-8.25 AU, ES: -0.12, p = 0.065). RWs in the time frame 6 to 8-hours and GKs in the 5 to 6-hours showed up to large decreases in PlayerLoad (ES: 1.3 to 2.54). LBs in the time frame 2 to 3-hours presented higher up to largely higher values.

***Random Effects and Model Fit:*** Random effects analysis revealed significant individual player variability (σ^2^ ranging from 0.25 to 62.54 across the models). The intraclass correlation coefficient (ICC) suggested that a substantial proportion of the variance could be attributed to inter-player differences (ranging from 0.62 to 0.83 across the models) (Table 2).

**TABLE 2:** Explanation of the result of the model

Predictors	Variables included in the model to predict the outcome.
**Estimates**	The coefficient values for each predictor, indicating the change in the outcome variable for one unit of change in the predictor.
**Estimate 95% CI**	5% Confidence Interval for the Estimates, providing a range within which the true value of the estimate is expected to fall.
**Es**	Magnitude of the effect, which can be interpreted as small, medium, or large according to conventional thresholds.
**95% CI (effect size)**	95% Confidence Interval for the Effect Size, suggesting the precision of the effect size estimate.
**p**	p-value indicating the probability that the observed effect or a more extreme one would occur if the null hypothesis were true
**Position**	Categorical variable representing different playing position.
**Group time**	Categorical variable representing different time interval.

**Random Effects**	
**σ2**	Variance at the level of the outcome.
**τ_00_**	Variance attributed to differences between players.
**ICC**	Proportion of the variance explained by differences between players.
**N**	Number of players in the study.
**Observations**	Total number of observations used in the model.
**Marginal R2 / Conditional R2**	Proportion of variance explained by the fixed effects alone (Marginal) and by the entire model including both fixed and random effects (Conditional).

**Analysis of Variance**	
**F value**	Test statistic for the variance analysis, indicating the ratio of the variance explained by the model to the unexplained variance.
**Pr(> F)**	p-value corresponding to the F statistic.
**Eta2_partial**	Partial eta-squared, a measure of effect size for the model.
**CI low / CI high**	Lower and upper bounds of the 95% Confidence Interval for the eta-squared statistic.

The marginal R^2^ values indicated that the fixed effects accounted for a considerable proportion of the variance in the observed outcomes (ranging from 0.511 to 0.777), while the conditional R^2^ values demonstrated a robust overall model fit when both fixed and random effects were considered (ranging from 0.823 to 0.926).

## DISCUSSION

Our results suggest that a player’s position significantly affects game demands, and that increases in cumulative playing time do not necessarily lead to substantial decreases in external load variables. GKs showed consistent and marked decreases across all measures, while wings exhibited a distinct high-speed running profile, covering more distance than other players. Contrary to our initial hypothesis, cumulative playing time did not significantly affect the external load of players, as indicated by non-significant P-values and very low ETA squared values.

This outcome can be attributed to effective substitution management strategies carried out by coaches, as demonstrated in soccer [20] and rugby [28]. Handball’s allowance for unlimited player rotation may further mitigate the impact of cumulative playing time on external load, enabling coaches to frequently substitute players and limit fatigue accumulation.

The minimal impact of cumulative playing time on player’s performance, particularly at higher intervals, might also be explained by the depth of successful teams’ rosters. Teams with more skilled players can distribute playing time more effectively, managing fatigue and maintaining performance throughout the whole tournament. As teams progress in competitions, they accumulate more playing time, yet the abundance of skilled players mitigates the potential adverse effects of this accumulation on individual performance.

For instance, in HSR wings are observed to accumulate more meters compared to findings from research conducted with male players [2, 5] (Figure 3). However, their meter accumulation does not increase as time progresses, likely due to rotational strategies employed during the game. The rotational strategy for wing players is attributed to their explosive nature, necessitating measures to minimize injury risk. These substitutions serve to effectively manage the load on these athletes [29]. Research in ice hockey by Spooner et al. [30] supports the idea that brief rest periods can lead to sufficient recovery. Given that handball is less physically demanding than ice hockey [31] these short breaks are likely sufficient for handball players to recover effectively.

While wings accumulate the highest HSR meters, CBs and RBs lead in the HIA (Figure 4), as wingers tend to be more static during positional play compared to these positions, which are more actively involved in generating play [6, 13].

Pacing strategies employed by players could further explain our findings (Figure 2). Studies [4, 21] have noted average external load level variations without significant changes at maximum intensity, suggesting that players strategically exert high intensity in crucial situations while conserving effort elsewhere. The increased pace in high-stakes matches, such as semi-finals and finals, may also contribute to this dynamic [32].

Situational variables, such as the current score and tournament standing [33], along with playing style [34], are also likely to influence these observations, similar to findings in football.

The variability within playing positions regarding cumulative playing time can also be explained by all or some the factors discussed above.

The high values of the coefficient when random effect (players) was included with a Conditional R^2^ between 0.82 and 0.92, highlights the influence of individual player profiles (Table 3). Factors like aerobic capacity, physical attributes, and playing style play a crucial role in performance [3, 35]. Individual characteristics, including size, body mass, and technical abilities, also contribute to performance variability [35, 36]. These attributes influence how players adapt to and tackle game challenges, exemplified by the contrasting playing styles of players such as Nora Mork from Norway and Cristina Neagu from Romania, who occupy the same position yet exhibit distinct approaches. Mork is more inclined to break through a defense, whereas Neagu prefers to shoot from afar.

**TABLE 3:** Performance metrics analysed

	Total distance					High speed running distance				
Predictors	Estimates	Estimate 95% CI	Es	95% CI	p	Estimates	Estimate 95% CI	Es	95% CI	p
(Intercept)	88.55	85.12 to 91.99	0.54	0.34 to 0.73	**< 0.001**	28.00	25.96 to 30.04	**1.33**	1.11 to 1.55	**< 0.001**
Position [RW]	-0.93	-4.79 to 2.93	-0.05	-0.27 to 0.17	0.635	0.20	-2.10 to 2.50	0.02	-0.23 to 0.27	0.865
Position [CB]	4.03	0.33 to 7.74	0.23	0.02 to 0.44	**0.033**	-12.38	-14.59 to -10.16	**-1.33**	-1.57 to -1.1	**< 0.001**
Position [LB]	-1.33	-4.92 to 2.26	-0.08	-0.28 to 0.13	0.466	-14.18	-16.32 to -12.03	**-1.53**	-1.76 to -1.3	**< 0.001**
Position [RB]	-1.00	-4.92 to 2.93	-0.06	-0.28 to 0.17	0.619	-11.74	-14.08 to -9.40	**-1.27**	-1.52 to -1.01	**< 0.001**
Position [PIV]	-5.80	-9.45 to -2.16	-0.33	-0.54 to -0.12	**0.002**	-11.87	-14.04 to -9.69	**-1.28**	-1.51 to -1.04	**< 0.001**
Position [GK]	-45.75	-49.42 to -42.08	**-2.62**	-2.82 to -2.41	**< 0.001**	-25.79	-27.98 to -23.61	**-2.78**	-3.02 to -2.54	**< 0.001**
Group_time [1 h–2 h]	-0.80	-4.04 to 2.43	-0.05	-0.23 to 0.14	0.625	-0.95	-2.87 to 0.97	-0.10	-0.31 to 0.1	0.332
Group_time [2 h–3 h]	-1.50	-4.88 to 1.88	-0.09	-0.28 to 0.11	0.384	-1.15	-3.16 to 0.86	-0.12	-0.34 to 0.09	0.262
Group_time [3 h–4 h]	-2.90	-6.06 to 0.27	-0.17	-0.35 to 0.02	0.073	-1.95	-3.84 to -0.07	-0.21	-0.41 to -0.01	**0.042**
Group_time [4 h–5 h]	-3.68	-7.16 to -0.20	-0.21	-0.41 to -0.01	**0.038**	-1.40	-3.47 to 0.67	-0.15	-0.37 to 0.07	0.184
Group_time [5 h–6 h]	-3.98	-8.42 to 0.46	-0.23	-0.48 to 0.03	0.079	-0.15	-2.79 to 2.50	-0.02	-0.3 to 0.27	0.913
Group_time [> 6 h]	-5.64	-12.22 to 0.94	-0.32	-0.7 to 0.05	0.093	-4.32	-8.25 to -0.40	-0.47	-0.89 to -0.04	**0.031**

	**Random Effects**					**Random Effects**				
σ2	26.16					8.63				
τ_00_	43.28 _Players_					15.57 _Players_				
ICC	0.62					0.64				
N	208 _Players_					208 Players				
Observations	1013					1013				
Marginal R^2^ / Conditional R^2^	0.777 / 0.916					0.720 / 0.900				
	Analysis of variance					Analysis of variance				

	**F value**	**Pr(> F)**	**Eta2_partial**	**CI_low**	**CI_high**	**F value**	**Pr(> F)**	**Eta2_partial**	**CI_low**	**CI_high**

Position	175.85	P < 0.0001***	0.84	0.81	1	123.44	P < 0.0001***	0.79	0.76	1
Group_Time	1.46	0.19	0.04	0	1	1.35	0.23	0.04	0	1

	**High intensity Action**					**PlayerLoad**				

**Predictors**	**Estimates Estimate 95% CI**	**Es**	**95% CI**	**p**	**Estimates**	**Estimate 95% CI**	**Es**	**95% CI**	**p**

(Intercept)	2.90	2.56 to 3.25	0.37	0.08 to 0.66	**< 0.001**	111.15	102.60 to 119.69	0.19	-0.1 to 0.48	**< 0.001**
Position [RW]	-0.18	-0.57 to 0.21	-0.15	-0.48 to 0.17	0.363	-4.39	-14.29 to 5.51	-0.15	-0.49 to 0.19	0.384
Position [CB]	0.31	-0.06 to 0.68	0.26	-0.05 to 0.57	0.102	15.79	6.28 to 25.29	0.54	0.21 to 0.86	**0.001**
Position [LB]	-0.05	-0.42 to 0.31	-0.05	-0.35 to 0.26	0.765	7.64	-1.60 to 16.89	0.26	-0.05 to 0.57	0.105
Position [RB]	0.32	-0.07 to 0.72	0.27	-0.06 to 0.6	0.110	11.26	1.24 to 21.29	0.38	0.04 to 0.72	0.028
Position [PIV]	-0.54	-0.91 to -0.18	-0.46	-0.76 to -0.15	**0.004**	8.78	-0.59 to 18.14	0.30	-0.02 to 0.62	**0.066**
Position [GK]	-2.38	-2.75 to -2.01	**-2.00**	-2.31 to -1.69	**< 0.001**	-54.21	-63.59 to -44.84	**-1.85**	-2.16 to -1.53**< 0.001**
Group_time [1 h-2 h]	-0.09	-0.42 to 0.23	-0.08	-0.35 to 0.19	0.572	-5.27	-13.28 to 2.75	-0.18	-0.45 to 0.09	0.198
Group_time [2 h-3 h]	0.11	-0.23 to 0.45	0.09	-0.19 to 0.37	0.531	-2.54	-11.01 to 5.93	-0.09	-0.37 to 0.2	0.556
Group_time [3 h-4 h]	-0.13	-0.45 to 0.18	-0.11	-0.38 to 0.16	0.413	-4.56	-12.53 to 3.41	-0.16	-0.43 to 0.12	0.262
Group_time [4 h-5 h]	-0.29	-0.64 to 0.06	-0.24	-0.54 to 0.05	0.104	-8.25	-17.01 to 0.50	-0.28	-0.58 to 0.02	0.065
Group_time [5 h-6 h]	-0.00	-0.45 to 0.44	0.00	-0.37 to 0.37	0.995	-2.58	-13.92 to 8.77	-0.09	-0.47 to 0.3	0.656
Group_time [> 6 h	-0.61	-1.27 to 0.05	-0.52	-1.07 to 0.04	0.069	-8.71	-25.78 to 8.35	-0.30	-0.88 to 0.28	0.317

	**Random Effects**					**Random Effects**				

σ2	0.25					62.54				
τ_00_	0.44 _Players_					313.49 _Players_				
ICC	0.64					0.83				
N	208 _Players_					207 _Players_				
Observations	1013					1007				
Marginal R^2^ / Conditional R^2^	0.511 / 0.823					0.556 / 0.926				
	Analysis of variance					Analysis of variance				

	**F value**	**Pr(> F)**	**Eta2_partial**	**CI_low**	**CI_high**	**F value**	**Pr(> F)**	**Eta2_partial**	**CI_low**	**CI_high**

Position	51.11	P < 0.0001***	0.61	0.54	1	52.51	P < 0.0001***	0.62	0.55	1
Group_Time	1.45	0.19	0.04	0	1	0.73	0.62	0.02	0	1

Our findings are consistent with prior research [2, 5], showing that wings accumulate the greatest distance and engage the most high-intensity actions (HIA) (Figure 4), but display lower PlayerLoad metrics (Figure 5). This phenomenon may stem from wings frequently remaining stationary during stabilized phases of play, in contrast to backs, who participate more in tactical movements [37], thus accruing a higher load. This observation is supported by additional studies on handball players [6, 15].

Regarding GKs, our findings support previous results [38] that highlight their specific needs and lower physical demands during games. This is normal because they do not tend to leave the goal area excessively, only in offensive inferiority actions or in offensive superiority actions (7 × 6) [13].

However, some methodological issues must be addressed. The varying definitions of playing time (e.g., effective playing time, time on the pitch) could lead to different interpretations, as discussed in studies on ice hockey [39], a sport with unlimited player rotation. Additionally, our study did not account for specialized players (offensive or defensive), who likely have different game demands than players participating in both phases [38].

This outcome of the research may be attributed to a variety of factors, including rigorous preparation and optimal fitness levels, alongside players’ strategic pacing. Additionally, the implementation of effective substitution management by coaches, facilitated by the rules allowing unlimited player rotation in handball, plays a crucial role. Teams with extensive rosters have the advantage of distributing playing time more efficiently, thereby effectively managing fatigue and sustaining performance levels throughout the tournament through strategic substitutions.

Furthermore, individual player attributes—ranging from aerobic capacity and physical traits to distinct playing styles—markedly impact performance and the capacity to navigate game challenges. This is particularly evident in the substantial variability observed across different playing positions.

## Practical applications

Effective player management is essential for coaches in team sports. Developing a strategic approach to substitutions, as emphasized in studies by García-Aliaga et al. [20] and Lorenzo-Martinez et al. [40], is crucial for securing game victories. Coaches must focus on players’ fitness and recovery needs, accurately assessing fitness levels to determine optimal recovery periods. Understanding each player’s capabilities, playing position requirements, body dimensions, and technical characteristics is vital for making informed decisions about player rotations and recovery durations.

The player’s position significantly affects the external load during the game. Specifically, goalkeepers (GKs) consistently show marked decreases in external load, demonstrating lower physical demands in comparison to outfield positions. In contrast, wing players are distinguished by their extensive coverage of distance and engagement in high-speed running, surpassing the requirements of other player positions. For wing players, specialized speed training is imperative due to their high-speed action demands.

Therefore, a targeted approach to training is necessary to work above and beyond the needs of the competitive load before the start of a match-intensive championship. Adding player management ensures that each player is optimally prepared to meet the specific demands of his role on the field.

At the same time, the control of the training load during the tournament must be taken into account as there will normally be few training sessions and these will be aimed more at recovery and match preparation training than at conditional work.

## CONCLUSIONS

The study demonstrates that a player’s position significantly influences the external load during a game, yet, contrary to our initial hypothesis, cumulative playing time does not substantially affect external load variables. Notably, athletes participating in critical stages of the EHF Women’s EURO 2022 exhibited no reductions in external load variables.

In conclusion, our research underscores the intricate relationship among player position, cumulative playing time, team composition, pacing strategies, and individual player characteristics in shaping external load and performance metrics in women’s handball. This multifaceted interplay suggests that achieving success and optimal performance in the sport requires a comprehensive approach that integrates team strategies with individual player development.

According to the data we have obtained, we believe that the key to optimally preparing the players is to ensure that, before the championship, the training sessions are highly specific to the external load that the players will face during the competition. This specific load will optimally enhance their performance while also helping to prevent injuries. It is important to note that in indoor sports, we can train above competitive demands during the training microcycle without the fatigue associated with outdoor sports.

In terms of optimal external load during competition, there are no specific values that can be defined. It will depend on the sporting outcomes, the players’ performance, and the reduction in injuries among them. Additionally, the style of play, the management of the squad for each match, as well as the substitutions made during the game and the final result, must also be taken into account.

The performance of the present study required the design of an integral and modular system based on ICT techniques and technologies such as sensors network, LPS, and big data analytics to process such a quantity of information coming from the championship.

## References

[1] Manchado C, Pueo B, Chirosa-Rios LJ, Tortosa-Martínez J. Time–motion analysis by playing positions of male handball players during the european championship 2020. Int J Environ Res Public Health. 2021 Mar 2; 18(6):1–15. 10.3390/ijerph18062787 PMC8002104 33801814

[2] Font R, Karcher C, Loscos-Fàbregas Tortosa-Martínez JE, Altarriba-Bartés A, Peña J, Vicens-Bordas J, Mesas JA, Irurtia A. The effect of training schedule and playing positions on training loads and game demands in professional handball players. Biol Sport. 2023; 40(3):857–866. 37398952 10.5114/biolsport.2023.121323 PMC10286613

[3] Impellizzeri FM, Marcora SM, Coutts AJ. Internal and External Training Load: 15 Years On Training Load: Internal and External Load Theoretical Framework: The Training Process. Int J Sports Physiol Perform. 2019; 14(2):270–3. 30614348 10.1123/ijspp.2018-0935

[4] Calleja-González J, Mallo J, Cos F, Sampaio J, Jones MT, Marqués-Jiménez D, et al. A commentary of factors related to player availability and its influence on performance in elite team sports. Front Sports Act Living. 2023 Jan 16; 4. 10.3389/fspor.2022.1077934 PMC9885271 36726395

[5] Font R, Karcher C, Reche X, Carmona G, Tremps V, Irurtia A. Monitoring external load in elite male handball players depending on playing positions. Biol Sport. 2021; 38(3):3–9. 34475629 10.5114/biolsport.2021.101123 PMC8329973

[6] Luteberget LS, Spencer M. High-intensity events in international women’s team handball matches. Int J Sports Physiol Perform. 2017; 12(1):56–61. 27071136 10.1123/ijspp.2015-0641

[7] Kniubaite A, Skarbalius A, Clemente FM, Conte D. Quantification of external and internal match loads in elite female team handball. Biol Sport. 2019; 36(4):311–6. 31938001 10.5114/biolsport.2019.88753 PMC6945049

[8] Carton-Llorente A, Lozano D, Gilart Iglesias V, Jorquera D, Manchado C. Worst-case scenario analysis of physical demands in elite men handball players by playing position through big data analytics. Biol Sport. 2023; 40(4):1219–27. 37867747 10.5114/biolsport.2023.126665 PMC10588589

[9] Zapardiel JC, Asín-Izquierdo I, Manchado C, Marcos-Jorquera D, Gilart-Iglesias V, Lozano D. Competitive profile analysis according to playing positions of female handball players during the European Championship 2020. Int J Perform Anal Sport. 2024; 1–19. 10.1080/24748668.2024.2333655

[10] Fleureau A, Lacome M, Buchheit M, Couturier A, Rabita G. Validity of an ultra-wideband local positioning system to assess specific movements in handball. Biol Sport. 2020; 37(4):351–7. 33343068 10.5114/biolsport.2020.96850 PMC7725040

[11] Luteberget LS, Spencer M, Gilgien M. Validity of the Catapult ClearSky T6 local positioning system for team sports specific drills, in indoor conditions. Front Physiol. 2018; 9:115. doi: 10.3389/fphys.2018.00115 29670530 PMC5893723

[12] Pérez Armendáriz ML, Spyrou K.E., Alcaraz P. Match demands of female team sports: a scoping review. Biol Sport. 2024; 41(1):175–199. 10.5114/biolsport.2024.129476 PMC10765441 38188119

[13] Manchado C, Pers J, Navarro F, Han A, Sung E, Platen P. Time-motion analysis in women’s team handball: Importance of aerobic performance. J Hum Sport Exerc. 2013; 8(2 Suppl):376–90.

[14] Luteberget LS, Trollerud HP, Spencer M. Physical demands of game-based training drills in women’s team handball. J Sports Sci. 2018 Mar 4; 36(5):592–8. 28508705 10.1080/02640414.2017.1325964

[15] Wik EH, Luteberget LS, Spencer M. Activity profiles in international women’s team handball using PlayerLoad. Int J Sports Physiol Perform. 2017 Aug 1; 12(7):934–42. 27967272 10.1123/ijspp.2015-0732

[16] Fullagar HH, Skorski S, Duffield R, Hammes D, Coutts AJ, Meyer T. Sleep and athletic performance: the effects of sleep loss on exercise performance, and physiological and cognitive responses to exercise. Sports Med. 2015 Feb;45(2):161-86. 25315456 10.1007/s40279-014-0260-0

[17] Yang J, Wu C, Zhou C, Zhang S, Leicht AS, Gomez MÁ. Influence of Match Congestion on Performances in the National Basketball Association. Front Psychol. 2021; 12:630769. doi: 10.3389/fpsyg.2021.630769 33679556 PMC7925613

[18] Carling C, McCall A, Le Gall F, Dupont G. The impact of short periods of match congestion on injury risk and patterns in an elite football club. Br J Sports Med. 2016 Jun;50(12):764-8. doi: 10.1136/bjsports-2015-095501. 26682867

[19] Page RM, Field A, Langley B, Harper LD, Julian R. The Effects of Fixture Congestion on Injury in Professional Male Soccer: A Systematic Review. Sports Med. 2023 Mar;53(3):667-685. doi: 10.1007/s40279-022-01799-5. 36527592 PMC9758680

[20] García-Aliaga A, Martín-Castellanos A, Marquina Nieto M, Muriarte Solana D, Resta R, López del Campo R, et al. Effect of Increasing the Number of Substitutions on Physical Performance during Periods of Congested Fixtures in Football. Sports. 2023; 11(2):25 36828310 10.3390/sports11020025 PMC9962594

[21] Doncaster G, White P, Svenson R, Page RM. The influence of fixture congestion on physical performance response to U23 soccer match-play. Res Sports Med. 2023 Jul-Dec;31(4):491-505. doi: 10.1080/15438627.2021.2001649. 34747292

[22] López-Valenciano A, Raya-González J, Garcia-Gómez JA, Aparicio-Sarmiento A, Sainz de Baranda P, De Ste Croix M, Ayala F. Injury Profile in Women’s Football: A Systematic Review and Meta-Analysis. Sports Med. 2021 Mar;51(3):423-442. doi: 10.1007/s40279-020-01401-w. 33433863

[23] Snyder BJ, Hutchison RE, Mills CJ, Parsons SJ. Effects of two competitive soccer matches on landing biomechanics in female division i soccer players. Sports. 2019; 7(11): 237. 31739531 10.3390/sports7110237 PMC6915335

[24] Sánchez-Migallón V, López-Samanes Á, Del Coso J, Navandar A, Aagaard P, Moreno-Pérez V. Effects of consecutive days of matchplay on maximal hip abductor and adductor strength in female field hockey players. BMC Sports Sci Med Rehabil. 2022 Jan 3;14(1):3. 34980243 10.1186/s13102-021-00394-x PMC8725242

[25] Esteves PT, Mikolajec K, Schelling X, Sampaio J. Basketball performance is affected by the schedule congestion: NBA back-to-backs under the microscope. Eur J Sport Sci. 2021; 21(1):26–35. 32172667 10.1080/17461391.2020.1736179

[26] Winter EM, Maughan RJ. Requirements for ethics approvals. J Sports Sci. 2009; 27(10):985–985. 19847681 10.1080/02640410903178344

[27] Batterham AM, Hopkins WG. Making meaningful inferences about magnitudes. Int J Sports Physiol Perform. 2006; 1(1):50–7. 19114737

[28] Michael I, Serpell BG, Colomer CM, Mara JK. Analysing the short-term impact of substitutes vs. starters in international rugby. Int J Sports Sci Coach. 2019 Oct 18; 14(5):667–74.

[29] Karcher C, Buchheit M. On-Court demands of elite handball, with special reference to playing positions. Sports Med. 2014; 44(6):797–814. 24682948 10.1007/s40279-014-0164-z

[30] Spooner TW, West AT, Willems MET. Effect of Substitution Time on Physical, Technical and Cognitive Performance in Sub-Elite Male Field Hockey Players. Int J Exerc Sci. 2023 Apr 1;16(6):497-512. 10.70252/BDLO6083 PMC10446958 37621709

[31] Vigh-Larsen JF, Mohr M. The physiology of ice hockey performance: An update. Scand J Med Sci Sports. 2024 Jan 29; 34(1). 10.1111/sms.14284 36517860

[32] Laxdal A, Ivarsson A, Sigurgeirsson O, Haugen T. Are the playoffs different from the regular season? A comparison of in-game statistics in Icelandic elite handball. Int J Perform Anal Sport. 2022 Sep 3; 22(5):649–655.

[33] Konefał M, Radzimiński Ł, Chmura J, Modrić T, Zacharko M, Padrón-Cabo A, Sekulic D, Versic S, Chmura P. The seven phases of match status differentiate the running performance of soccer players in UEFA Champions League. Sci Rep. 2023 Apr 24; 13(1):6675. 37095241 10.1038/s41598-023-33910-9 PMC10126199

[34] Calder A, Gabbett T. Influence of Tactical Formation on Average and Peak Demands of Elite Soccer Match-Play. Int J Strength Cond. 2022; 2(1).

[35] Martínez-Rodríguez A, Martínez--Olcina M, Hernández-García M, Rubio-Arias J, Sánchez-Sánchez J, Sánchez-Sáez JA. Body composition characteristics of handball players: systematic review. Arch Med Deporte. 2020; 37(1):52–61.

[36] González-Haro PJ, Gómez-Carmona CD, Bastida-Castillo A, Rojas-Valverde D, Gómez-López M, Pino-Ortega J. Analysis of playing position and match statusrelated differences in external load demands on amateur handball: A case study. Rev Bras Cineantropom Desempenho Hum. 2020; 22(June):1–13.

[37] Rogulj N, Vuleta D, Milanović D, Čavala M, Foretić N. The efficiency of elements of collective attack tactics in handball. Kinesiol Sloven. 2011; 17(1):5–14.

[38] Lefèvre T, Guignard B, Karcher C, Reche X, Font R, Komar J. A deep dive into the use of local positioning system in professional handball: Automatic detection of players’ orientation, position and game phases to analyse specific physical demands. PLoS One. 2023 Aug 16; 18(8):e0289752. 37585452 10.1371/journal.pone.0289752 PMC10431627

[39] Staunton CA, Björklund G. A Framework for the Standardization of Game Analysis in Ice Hockey. Int J Sports Physiol Perform. 2023 May 1; 18(5):458–64. 36889324 10.1123/ijspp.2022-0260

[40] Lorenzo-Martínez M, Rein R, Garnica-Caparrós M, Memmert D, Rey E. The Effect of Substitutions on Team Tactical Behavior in Professional Soccer. Res Q Exerc Sport. 2022 Apr 3; 93(2):301–9. 33054664 10.1080/02701367.2020.1828563

